# The Comparison of Different Stretching Intensities on the Range of Motion and Muscle Stiffness of the Quadriceps Muscles

**DOI:** 10.3389/fphys.2020.628870

**Published:** 2021-01-13

**Authors:** Masatoshi Nakamura, Shigeru Sato, Yuta Murakami, Ryosuke Kiyono, Kaoru Yahata, Futaba Sanuki, Riku Yoshida, Taizan Fukaya, Kosuke Takeuchi

**Affiliations:** ^1^Institute for Human Movement and Medical Sciences, Niigata University of Health and Welfare, Niigata, Japan; ^2^Department of Physical Therapy, Niigata University of Health and Welfare, Niigata, Japan; ^3^Department of Rehabilitation, Kyoto Kujo Hospital, Kyoto, Japan; ^4^Department of Physical Therapy, Faculty of Rehabilitation, Kobe International University, Hyogo, Japan

**Keywords:** high-intensity stretching, shear elastic modulus, stretch tolerance, visual analog scale, static stretching

## Abstract

Muscle strain is one of the most frequent sports injuries, having the rectus femoris (RF) muscle as the reported preferred site of quadriceps muscle strain. The decrease muscle stiffness could be an effective RF muscle strain prevention. In recent studies, a high-intensity static stretching intervention decreased passive stiffness, though no study has investigated on the effect of the different static stretching intervention intensities on quadriceps muscle stiffness. The purpose of this study was to investigate the three different quadriceps muscle stiffness intensities (120 vs. 100 vs. 80%). Eighteen healthy, sedentary male volunteers participated in the study and randomly performed three intensities. The static stretching intervention was performed in knee flexion with 30° hip extension. Three 60-second stretching intervention with a 30-second interval were performed at each stretching intensity. We measured knee flexion range of motion and shear elastic modulus of the RF muscle used by ultrasonic shear-wave elastography before and after the static stretching intervention. Our results showed that the knee flexion range of motion was increased after 100% (*p* < 0.01) and 120% intensities (*p* < 0.01) static stretching intervention, not in 80% intensity (*p* = 0.853). In addition, our results showed that the shear elastic modulus of the RF muscle was decreased only after 100% intensity static stretching intervention (*p* < 0.01), not after 80% (*p* = 0.365), and 120% intensities (*p* = 0.743). To prevent the quadriceps muscle strain, especially the RF muscle, 100%, not 120% (high) and 80% (low), intensity stretching could be beneficial in sports setting application.

## Introduction

One of the most frequent sports injuries and the third most common in a survey of injuries occurring during the Olympic Games in Rio de Janeiro is muscle strain, after sprain/ligament rupture and contusion/hematoma/bruise (Soligard et al., 2017). Regarding incidence of muscle strain, the hamstrings, quadriceps, and triceps muscles are the most frequently involved (Garrett, 1996; Orchard et al., 2020). In a study, which used MRI, the rectus femoris (RF) muscle was the reported preferred muscle strain site of the quadriceps muscle (Cross et al., 2004). Moreover, the lower muscle stiffness could contribute in sport injury prevention (Watsford et al., 2010; Pickering Rodriguez et al., 2017). Although not all sports injuries can be prevented, stretching interventions have been shown to prevent the muscle strains (Mchugh and Cosgrave, 2010). Taken together, these results suggested that the decrease in muscle stiffness of the quadriceps muscle, especially the RF muscle, could be beneficial, preventing quadriceps muscle strains after stretching intervention.

Previous studies showed that muscle stiffness in the plantar flexors (Nakamura et al., 2014, 2017, 2019), hamstrings (Umegaki et al., 2015; Miyamoto et al., 2017), and quadriceps (Caliskan et al., 2019) were decreased immediately after a static stretching intervention. Interestingly, while recent studies focused on the stretching intensity, Kataura et al. (2017) investigated on the effect of three different stretching intensities on the hamstring muscle-tendon unit passive stiffness, with results showing the effectiveness of a high-intensity static stretching intervention in increasing range of motion (ROM) and decreasing passive stiffness of the muscle-tendon unit (Kataura et al., 2017). Similarly, other studies have shown that high-intensity static stretching was effective for decreasing passive stiffness of the hamstring muscle-tendon unit even if with a short stretching intervention duration (Fukaya et al., 2020a; Takeuchi and Nakamura, 2020). In addition, Fukaya et al. (2020a) compared the effect of high- and low-intensity static stretching interventions with the same total stretching load on muscle stiffness of the medial gastrocnemius muscle, which showed larger changes in dorsiflexion ROM and muscle stiffness after a high-volume static stretching intervention compared with a low-intensity static stretching intervention even with the same stretching load. Thus, previous studies suggested that a high-intensity static stretching intervention could decrease muscle stiffness in the quadriceps muscle, but, to the best of our knowledge, no study investigated on the different stretching intensities, especially high-intensity stretching on the quadriceps muscle; hence, high-intensity stretching effectiveness in the quadriceps muscle is also unclear.

The purpose of this study was to investigate the effect of three different intensities (120, 100, and 80%) on the knee flexion ROM and stiffness of the RF, vastus lateralis (VL), and medialis (VM) muscles. We hypothesized that the higher static stretching intensity was effective in the knee flexion ROM changes and muscle stiffness, based on the previous studies (Kataura et al., 2017; Takeuchi and Nakamura, 2020).

## Materials and Methods

### Experimental Design

We used a randomized repeated measures experimental design to compare the effects of three different static stretching intensities (120 vs. 100 vs. 80%) (Kataura et al., 2017) on the ROM, shear elastic modulus of the quadriceps muscle (RF, VL, and VM), and stretching pain in the dominant leg (ball kicking preference). Participants visited the laboratory for three times with a >72 h interval and performed three stretching intensities randomly. In this study, the knee flexion ROM and shear elastic modulus of the RF, VL, and VM muscles were assessed before (PRE) and immediately after 60 seconds for three stretching intervention repetitions (POST). In addition, the quadriceps muscle pain magnitude was assessed during each stretching using a visual analog scale.

### Participants

The participants were 18 healthy, sedentary male volunteers (age: 22.7 ± 2.8 years; height: 169.1 ± 4.2 cm; body mass: 63.6 ± 6.6 kg) and excluded those with neuromuscular disease or lower extremity musculoskeletal injury history. During the experimental period, all participants were instructed not to perform resistance and flexibility training of the lower limbs. All participants were informed detailed information on the study procedures and purpose, and they provided written informed consents. The study was approved by the Ethics Committee of the Niigata University of Health and Welfare, Niigata, Japan, (Procedure #17677), and complied with the Declaration of Helsinki requirements. The sample size required for a two-way repeated analysis of variance (ANOVA) [effect size = 0.25 (medium), α error = 0.05, and power = 0.80] was calculated using G^∗^power 3.1 software (Heinrich Heine University, Düsseldorf, Germany) based on the previous study, and the required number of participants was 18 participants in this study.

### Assessment of Knee Flexion Range of Motion

Subjects positioned on a 90° flexed hip and the knee joints of the non-dominant leg and 30° hip joint of the on the dominant side, as the reference limb position. Afterward, the investigator flexed the knee joint passively from the reference limb position to the knee flexion angle just before the subjects started to feel discomfort or pain (Akagi and Takahashi, 2013; Sato et al., 2020). The knee flexion ROM was measured using a goniometer twice, using the average value for further analysis.

### Assessment of the Shear Elastic Modulus of the Quadriceps Muscle

In this study, we measured the shear elastic modulus of the RF, VL, and VM muscles using the ultrasonic shear-wave elastography (Aixplorer Supersonic Imagine, Aix-en-Provence, France) with a SL10-2 linear prove. The participants were lying on the treatment bed at neutral hip joint position with 90° flexed hip and knee joint, where the shear elastic moduli of the RF, VL, and VM muscles were measured at the midpoint, 60 and 80% distal between the anterior superior iliac spine and the proximal end of the patella, respectively. The size of the region of interest was 10 × 20 mm^2^ and set near the each muscle center, with an analysis area of a 5-mm-diameter circle at the center of the stiffer region (Saeki et al., 2019). Long-axis elastographic images were obtained in two times. Based on previous studies (Hirata et al., 2016; Nakamura et al., 2019), the shear elastic modulus was calculated by dividing the obtained Young’s modulus by three. The average shear elastic modulus value obtained from the duplicate elastographic images was used for analysis.

Prior to the study, we confirmed that the shear elastic modulus measurement’s test-retest reliability for the RF, VL, and VM muscles was determined by coefficient variation and intraclass correlation coefficient using six legs in three healthy young males. The coefficient variation for shear elastic moduli of the RF, VL, and VM muscles were 6.2 ± 3.2, 9.7 ± 9.1, and 1.6 ± 1.1%, respectively, with intraclass correlation coefficient (1, 2) for the measurements of 0.876, 0.909, and 0.960, respectively.

### Assessment of Stretching Pain

The knee extensor muscle magnitude was assessed during each stretching intervention using a visual analog scale with a 100-mm continuous line with “not sore at all” on one side (0 mm) and “very, very sore” on the other (100 mm). Stretching pain assessments were performed during each stretching intervention for three times.

### Static Stretching Intervention Maneuver

The static stretching intervention was performed in a similar fashion with the knee flexion ROM assessment. Three different intensities (120, 100, and 80%) were calculated based on the knee flexion ROM in the PRE value in each condition. Specifically, in 120% intensity condition, the angle of stretching intervention was set to 1.2 times of the knee flexion ROM at PRE value. Three 60-second stretching interventions with a 30-second interval each intensity were performed, which were defined to the same knee flexion angle in each condition. Participants were instructed to be relaxed and raise their torso upright during stretching intervention.

### Statistical Analyses

SPSS (version 24.0; SPSS Japan Inc., Tokyo, Japan) was used for statistical analysis. For the knee flexion ROM and shear elastic modulus at PRE values, a one-way repeated measure analysis of variance (ANOVA) was performed to clarify the differences among the three intensities. For the knee flexion ROM and shear elastic modulus, we performed a two-way repeated measure ANOVA [time (PRE vs. POST) and stretching intensity condition (120 vs. 100 vs. 80%)] to analyze the interaction and main effect. If there was a significant interaction effect, we performed a paired *t*-test to compare the PRE and POST values and used the Bonferroni multiple comparison test to determine significant differences in the stretching intensity conditions. Effect size (ES) was calculated as a difference in the mean value between PRE and POST divided by the pooled SD (Cohen, 1988). ES of 0.00–0.19 was considered trivial, 0.20–0.49 was small, 0.50–0.79 was moderate, and ≥0.80 was large. For stretching pain, we performed a two-way repeated measure ANOVA [time (first bout vs. second bout vs. third bout) and stretching intensities condition (120 vs. 100 vs. 80%)] to analyze the interaction and main effect. If there was a significant interaction effect, we used the one-way repeated ANOVA and the Bonferroni multiple comparison test to determine significant differences among the times in each condition and stretching intensity. We assumed statistically significant differences of *p* < 0.05 at an alpha level and indicated descriptive data as means ± standard deviation.

## Results

### Comparison of PRE Values Among the Three Static Stretching Intensity Conditions

For the knee flexion ROM and shear elastic modulus of the RF, VL, and VM muscle, a one-way repeated ANOVA revealed no significant differences among the three stretching intensity conditions (*p* = 0.787, *F* = 0.463, η_*p*_^2^ = 0.044, *p* = 0.668, *F* = 0.409, η_*p*_^2^ = 0.023, *p* = 0.671, *F* = 0.403, η_*p*_^2^ = 0.023, and *p* = 0.983, *F* = 0.017, η_*p*_^2^ < 0.01, respectively).

### Changes in the Knee Flexion ROM Before and After a Static Stretching Intervention

**FIGURE 1:**
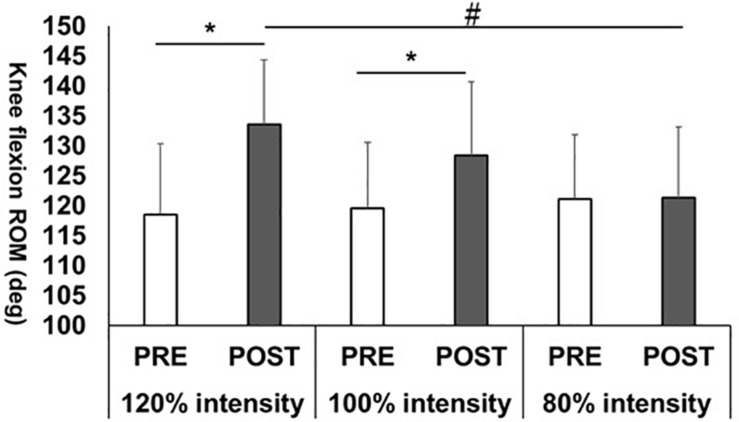
Knee flexion range of motion (ROM) changes before (PRE) and after (POST) stretching intervention.*p < 0.01, significant difference between PRE and POST; ^#^*p* < 0.01, significant difference between 120 and 80% intensities.

The changes in the knee flexion ROM before and after stretching intervention in all stretching intensity conditions were shown in Figure 1. The two-way repeated ANOVA showed the significant interaction effect (*p* < 0.01, *F* = 40.6, η_*p*_^2^ = 0.85). Regarding with comparison between PRE and POST values in each stretching intensity, paired *t*-test showed an increased the knee flexion ROM after 120% (*p* < 0.01, *d* = 1.33, 95% confidence interval, 12.5–17.6) and 100% stretching intervention intensities (*p* < 0.01, *d* = 0.75, 95% confidence interval, 5.6–11.9) with no significant change at 80% intensity (*p* = 0.853, *d* = 0.02, 95% confidence interval, −2.0–2.4).

Regarding with comparison between stretching intensities in POST, one-way repeated ANOVA showed a significant effect for the knee flexion ROM (*p* < 0.01, *F* = 7.15, η_*p*_^2^ = 0.296). In *post hoc* test, the Bonferroni multiple comparison test revealed a significantly higher knee flexion ROM value in 120% intensity than in 80% intensity value (*p* < 0.01, 95% confidence interval, 6.5–17.9) and showed no significant differences between 120 and 100% intensities (*p* = 0.429, 95% confidence interval, −3.9–14.6) or 100 and 80% intensities (*p* = 0.273, 95% confidence interval, −3.3 to 17.0).

### Changes in the Shear Elastic Moduli of the RF, VL, and VM Muscles Before and After the Static Stretching Intervention

**FIGURE 2:**
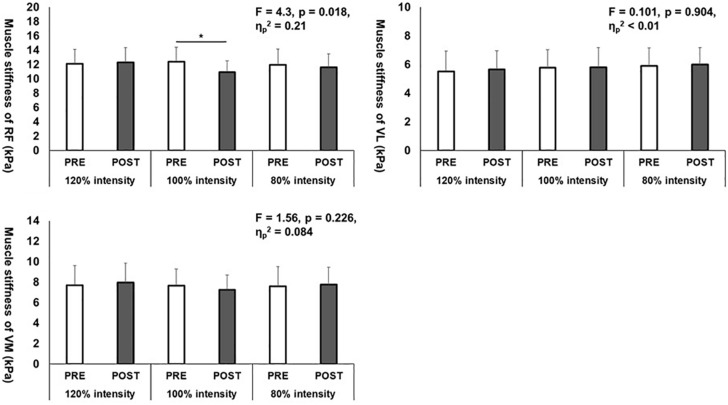
Shear elastic moduli changes of the rectus femoris (RF), vastus lateralis (VL), and vastus medialis (VM) muscles before (PRE) and after (POST) three different stretching intervention intensities. *p < 0.05, significant difference between PRE and POST.

The shear elastic modulus results were shown in Figure 2. A two-way repeated ANOVA showed the significant interaction effect in the shear elastic modulus of the RF muscle (*p* = 0.023, *F* = 4.53, η_*p*_^2^ = 0.21), and paired *t*-test revealed only significant difference between PRE and POST values in 100% intensity (*p* < 0.01, *d* = 0.83, 95% confidence interval, −6.7 to −2.7), not in 120% (*p* = 0.743, *d* = 0.11, 95% confidence interval, −2.3 to 3.7) and 80% intensities (*p* = 0.365, *d* = 0.17, 95% confidence interval, −3.5–1.3). However, there were no significant interaction effects in the shear elastic moduli of the VL and VM muscles (*p* = 0.101, *F* = 4.53, η_*p*_^2^ = 0.21; *p* = 0.226, *F* = 1.56, η_*p*_^2^ = 0.084, respectively) and main effect of time (*p* = 0.426, *F* = 0.665, η_*p*_^2^ = 0.038; *p* = 0.982, *F* = 0.001, η_*p*_^2^ > 0.01, respectively).

### Changes in Stretching Pain During a Static Stretching Maneuver

The results of stretching pain changes in three intensities were shown in Table 1. A two-way repeated ANOVA showed the significant interaction effect of the stretching pain magnitude (*p* < 0.01, *F* = 4.04, η_*p*_^2^ = 0.192). A one-way repeated ANOVA showed the main significant effects when the times in all stretching conditions were compared (120% intensity: *p* < 0.01, *F* = 7.19, η_*p*_^2^ = 0.297; 100% intensity: *p* < 0.01, *F* = 8.82, η_*p*_^2^ = 0.342; 80% intensity: *p* = 0.047, *F* = 3.35, η_*p*_^2^ = 0.165). The *post hoc* test revealed a significant decrease in the stretching pain during the third bout as compared to the first and second bouts in 120 and 100% intensities, while no significant differences were found in 80% intensity condition.

**TABLE 1:** Stretching pain changes during three stretching interventions in three different stretching intensities.

	First bout	Second bout	Third bout
120% intensity	59.5 ± 19.6^†‡^	55.8 ± 18.0^†‡^	48.8 ± 17.2*^#†‡^
100% intensity	13.5 ± 6.8^‡^	11.8 ± 7.1^‡^	9.5 ± 6.9*^#‡^
80% intensity	1.7 ± 3.5	0.3 ± 1.4	0.1 ± 0.5

Data presented as mean ± standard deviation. *significant difference in the first bout (*p* < 0.05); ^#^significant difference in the second bout (*p* < 0.05); ^†^significant difference at 100% intensity (*p* < 0.05); ^‡^significant difference at 80% intensity (*p* < 0.05).

A one-way repeated ANOVA showed the significant main effects when all stretching conditions in each time were compared (first bout: *p* < 0.01, *F* = 113.4, η_*p*_^2^ = 0.87; second bout: *p* < 0.01, *F* = 120.2, η_*p*_^2^ = 0.876; third bout: *p* < 0.01, *F* = 117.4, η_*p*_^2^ = 0.873). In all three stretching interventions, the *post hoc* test revealed that stretching pain in 120% intensity was significantly higher than those in 100 and 80% intensities, and the stretching pain in 100% intensity was significantly higher than that in 80% intensity.

## Discussion

In this study, we investigated the effect of three static stretching intensities on the knee flexion ROM and RF, VL, and VM muscle stiffness. Our results showed that the knee flexion ROM and muscle stiffness of RF were significantly reduced at 120 and 100% intensities and 100% intensity, respectively. Although there were some studies investigating high-intensity static stretching on the hamstring muscle-tendon unit (Kataura et al., 2017; Fukaya et al., 2020b; Takeuchi and Nakamura, 2020) and the medial gastrocnemius (Fukaya et al., 2020a), this is the first paper to investigate the effect of a high-intensity static stretching on knee flexion ROM and each muscle composing the quadriceps muscle.

Our results revealed a significantly increased knee flexion ROM after both 120 and 100% intensities but found no significant change after 80% intensity. In addition, the comparison of POST values showed that only 120% intensity was significantly higher than 80% intensity. These results showed that high-intensity stretching (120% intensity) was more beneficial for increasing the knee flexion ROM than normal stretching intensity (100% intensity) or low-intensity stretching (80% intensity). Recent studies suggested that an increased mechanism in ROM could be involved with stretch tolerance change, the sensation of stretching of the subject (Weppler and Magnusson, 2010; Kay et al., 2015; Freitas et al., 2018). In this study, the stretching pain during the static stretching intervention decreased gradually in 120 and 100% intensities, while no significant change is seen in 80% intensity, being consistent with the previous study (Kataura et al., 2017). Therefore, stretch tolerance changes could contribute in increasing the knee flexion ROM in 120 and 100% intensities, but not in 80% intensity where no significant stretch tolerance change is observed, which could not increase the knee flexion ROM. Furthermore, our results showed a higher stretching pain in 120% intensity compared to the 100 and 80% intensities. Despite not measuring passive torque at end ROM as the stretch tolerance index, significantly larger increase in the knee flexion ROM in 120% intensity than in 100 and 80% intensities is possible due to the large change in stretch tolerance.

In 100% intensity, the shear elastic modulus of the RF muscle was decreased significantly after static stretching intervention, with no significant changes in the shear elastic moduli in the VL and VM muscles, which could be related to the structural difference between the monoarticular and biarticular muscles. Since we adopted the static stretching maneuver as passive knee flexion with 30° extension of the hip joint (Figure 1), compared to the VL and VM muscles that are monoarticular muscles, RF, and a biarticular muscle, was considered to be the most stretched, resulting in the shear elastic modulus decrease. Also, Umegaki et al. (2015) investigated the effect of static stretching on the hamstring muscle, which showed significant decreases in the shear elastic moduli of the biceps femoris, semitendinosus, and semimembranosus muscles and larger decrease in the shear elastic modulus of the semitendinosus muscle than the biceps femoris and semimembranosus muscles (Umegaki et al., 2015). Thus, in this study, the effect of stretching intervention being different even in the muscle with the same action and among the quadriceps muscles is possible. Therefore, an investigation on the effect of the different stretching techniques on shear elastic modulus of each muscle composing the quadriceps muscle is needed.

In contrast to our hypothesis, where the shear elastic modulus of each muscle composing the quadriceps was decreased after 120% intensity, our results showed no significant changes in the shear elastic moduli of the RF, VL, and VM muscles after 120% intensity static stretching intervention. As the stretching intensities increases, the tension applied to the muscle increases, which assumes a larger decrease in muscle stiffness. However, stretching pain in 120% intensity is a very high value (first bout: 59.5 ± 19.6 mm; second bout: 55.8 ± 18.0 mm; third bout: 48.8 ± 17.2 mm, respectively). Apostolopoulos et al. (2015) investigated the effect of different stretching intervention intensities on inflammatory response and showed a significant increase in C-reactive protein induced by high-intensity stretching, potentially causing inflammation. In addition, previous study reported that sympathetic nerve activity is activated following pain and discomfort levels (Shiro et al., 2012). In this study, inflammatory response and sympathetic nerve activity changes are unknown, which might have interfered with the effects of muscle stiffness decrease in the quadriceps muscle. In addition, previous studies showed that a high-intensity static stretching intervention decreases passive stiffness of the hamstring muscle-tendon unit (Kataura et al., 2017; Fukaya et al., 2020b; Takeuchi and Nakamura, 2020) and muscle stiffness of the medial gastrocnemius (Fukaya et al., 2020a). The discrepancy between our study and previous studies could be related with the difference in target muscle, suggesting a possible difference in high-intensity static stretching intervention effects among the muscles. Therefore, the differences in static stretching intervention effect among the muscles, including the changes in inflammatory response and sympathetic nerve activity, need clarification.

In practical application, as mentioned above, the RF muscle was the reported preferred muscle strain site of the quadriceps muscle (Cross et al., 2004). Stretching intervention could possibly prevent muscle strains (Mchugh and Cosgrave, 2010). Our results revealed that 100% intensity static stretching intervention could increase the knee flexion ROM and in RF muscle stiffness, contributing in preventing RF muscle strain. Conversely, there was no significant change in the RF muscle stiffness after 120% static stretching intervention intensity, despite its effectiveness in the hamstring muscle (Kataura et al., 2017; Fukaya et al., 2020b; Takeuchi and Nakamura, 2020) and medial gastrocnemius muscle stiffness (Fukaya et al., 2020a). Our results suggested that 120% intensity, i.e., high-intensity stretching may not be beneficial for the quadriceps muscle when decreasing muscle stiffness. Since previous studies showed that a time dependent relationship between stretching duration and change in ROM or passive stiffness, is present (Nakamura et al., 2013; Thomas et al., 2018), in the future, determining the stretching time required to decrease muscle stiffness and whether or not stretching intervention can prevent muscle strains in the sports field is needed.

There were some limitations in this study. We only investigated the acute effect of stretching intervention, and whether or not high-intensity stretching intervention can prevent muscle strains in the sports field. Therefore, future studies are needed to investigate the long-term high-intensity stretching intervention effect and whether or not stretching intervention can prevent muscle strains in the sports field.

## Conclusion

We investigated the effect of different stretching intensities (120 vs. 100 vs. 80%) on the knee flexion ROM and shear elastic modulus of the quadriceps muscle (RF, VL, and VM). Our results showed that 120% intensity was the most effective in increasing the knee flexion ROM, whereas 100% intensity was effective in decreasing the shear elastic modulus of the RF muscle. To prevent quadriceps muscle strain, especially in the RF muscle, 100% intensity stretching, not 120% (high) or 80% (low), could be beneficial in sports setting application.

## Data Availability Statement

The raw data supporting the conclusions of this article will be made available by the authors, without undue reservation.

## Ethics Statement

The studies involving human participants were reviewed and approved by the Ethics Committee of the Niigata University of Health and Welfare, Niigata, Japan, (Procedure #17677). The patients/participants provided their written informed consent to participate in this study.

## Author Contributions

MN contributed to study design and data collection and drafted and critically revised the manuscript. SS, YM, RK, KY, FS, and RY contributed to data collection and made critical revisions to the manuscript. TF and KT contributed to study design, data analysis, and made critical revisions to the manuscript. All authors approved the final version of the manuscript and agreed to be accountable for all aspects of the work.

## Conflict of Interest

The authors declare that the research was conducted in the absence of any commercial or financial relationships that could be construed as a potential conflict of interest.
